# Supplementary material for: Pyridine nucleotide redox potential in coronary smooth muscle couples myocardial blood flow to cardiac metabolism
Source: Nat Commun. 2022 Apr 19;13:2051. doi: 10.1038/s41467-022-29745-z (PMC9018695; doi:10.1038/s41467-022-29745-z)
Supplement: Supplementary file 1 — Supplementary Information [file 41467_2022_29745_MOESM1_ESM.pdf]

Supplementary Information

Pyridine nucleotide redox potential in coronary smooth muscle couples myocardial blood flow to cardiac metabolism

Marc M. Dwenger[1], Sean M. Raph[1], Michelle L. Reyzer[2], M. Lisa Manier[2], Daniel W. Riggs[1], Zachary B. Wohl[1], Vahagn Ohanyan[3], Gregory Mack,[3] Thomas Pucci,[3] Joseph B. Moore[1], Bradford G. Hill[1], William M. Chilian[3], Richard M. Caprioli[2], Aruni Bhatnagar[1], Matthew A. Nystoriak[1]

[1]Department of Medicine, Division of Environmental Medicine, Diabetes and Obesity Center, University of Louisville, Louisville, KY 40202 USA; [2]Department of Biochemistry, Vanderbilt University School of Medicine, Nashville, Tennessee 37235 USA; [3]Department of Integrative Medical Sciences, Northeast Ohio Medical University, Rootstown Ohio 44272 USA.

Supplementary Figures and Tables

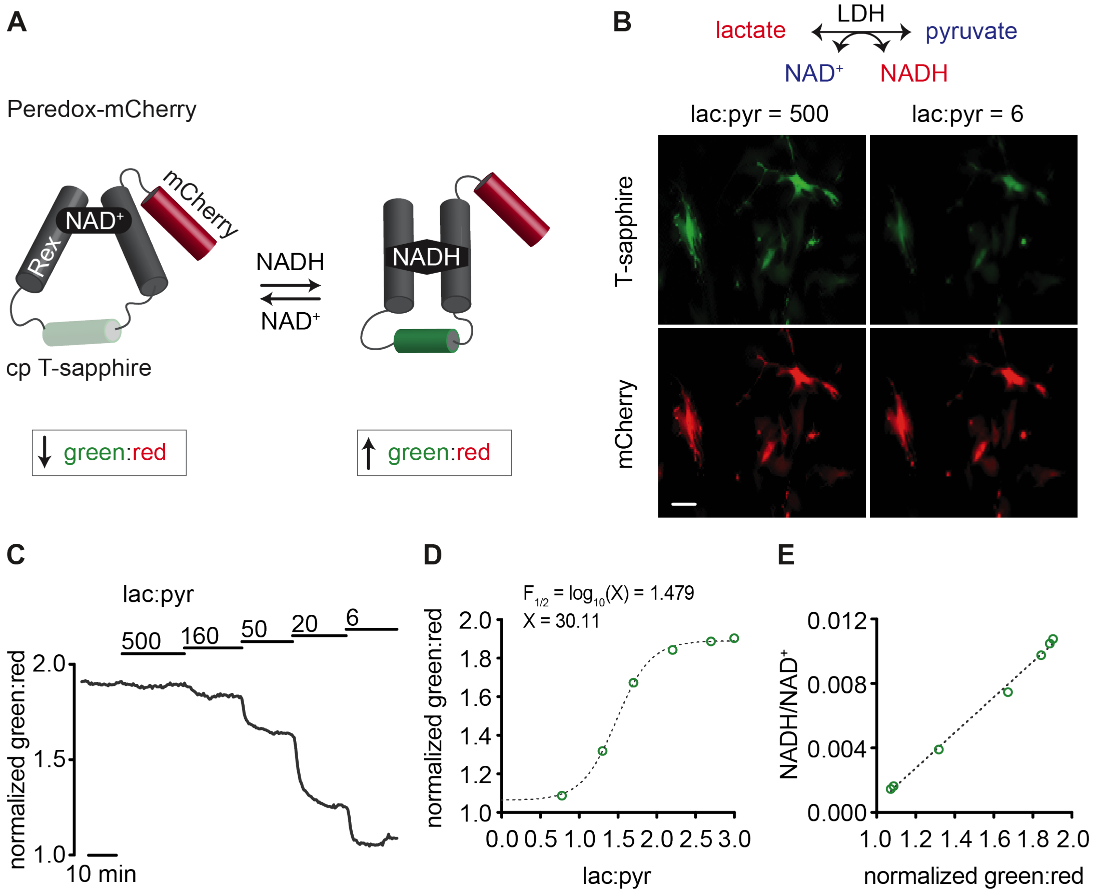

Supplementary Figure 1: Measurement of cytosolic NADH:NAD$^+$ in arterial smooth muscle cells. (**A**) Schematic representation of peredox-mCherry NADH:NAD$^+$-sensitive fluorescent biosensor. Peredox-mCherry is a modified bacterial redox-sensitive transcriptional repressor (i.e., Rex) that consists of circularly permutated T-sapphire and mCherry (see ref [1]). Upon binding of NADH, T-sapphire fluorescence is enhanced. No change in fluorescence is observed with mCherry, enabling normalization to biosensor expression via red fluorescence. (**B**) Representative fluorescence images showing T-sapphire and mCherry fluorescence in aortic vascular smooth muscle cells expressing peredox-mCherry in the presence of external lactate:pyruvate (lac:pyr) of 500 or 6. Scale bar represents 50 µm. Experiment was repeated three times with similar results. (**C-E**) Green:red fluorescence intensities (normalized to minimum ratio obtained in 10 mM pyruvate) in the presence of lac:pyr of 500-6 (*C*), mean green:red over the tested range of lac:pyr ratios (log scale), and mean cytosolic NADH:NAD$^+$ vs. green:red fluorescence, estimated using the protocol described in [2]. n = 87 cells, 3 independent experiments.

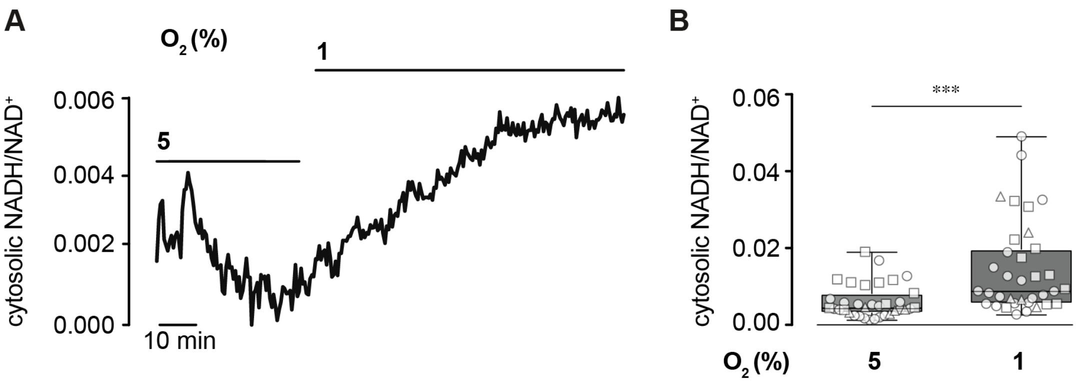

Supplementary Figure 2: Increased NADH:NAD$^+$ in arterial smooth muscle cells in the presence of low O_2. (**A**) Representative recording of cytosolic NADH:NAD$^+$ in an arterial myocyte in the presence of 5% and 1% O_2. NADH:NAD$^+$ in the presence of 10 mM lactate and 10 mM pyruvate, measured at the end of the experiment, were 0.0043 and 0.0015, respectively. (**B**) Box and whiskers plot (line: median, box: 25th to 75th percentile, whiskers: min and max) showing summarized NADH:NAD$^+$ in arterial myocytes in the presence of 5% and 1% O_2. n = 35 cells, 3 independent experiments (denoted by symbols). ***p = 4.65 x 10^{-8} (two-sided Wilcoxon matched pairs signed rank test).

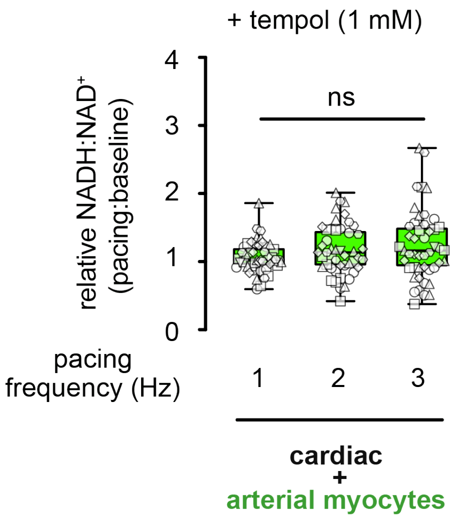

Supplementary Figure 3: Frequency-dependent increases in arterial myocyte NADH:NAD$^+$ are blunted in the presence of tempol. Box and whiskers plots (line: median, box: 25th to 75th percentile, whiskers: min and max) summarizing fold-change in NADH:NAD$^+$ in arterial myocytes in arterial/cardiac myocyte co-cultures electrically paced in the presence of 1 mM 4-hydroxy TEMPO (tempol). N = 47 cells from 6 independent experiments (denoted by symbols). Ns: $p = 0.0864$; arterial/cardiac myocyte vs. arterial/cardiac myocyte + tempol: 1 Hz, $p = 0.005$, 2 Hz, $p = 0.033$, 3 Hz, $p = 0.029$. Linear mixed models were used to analyze differences in log-transformed NADH:NAD$^+$ across frequencies and between groups.

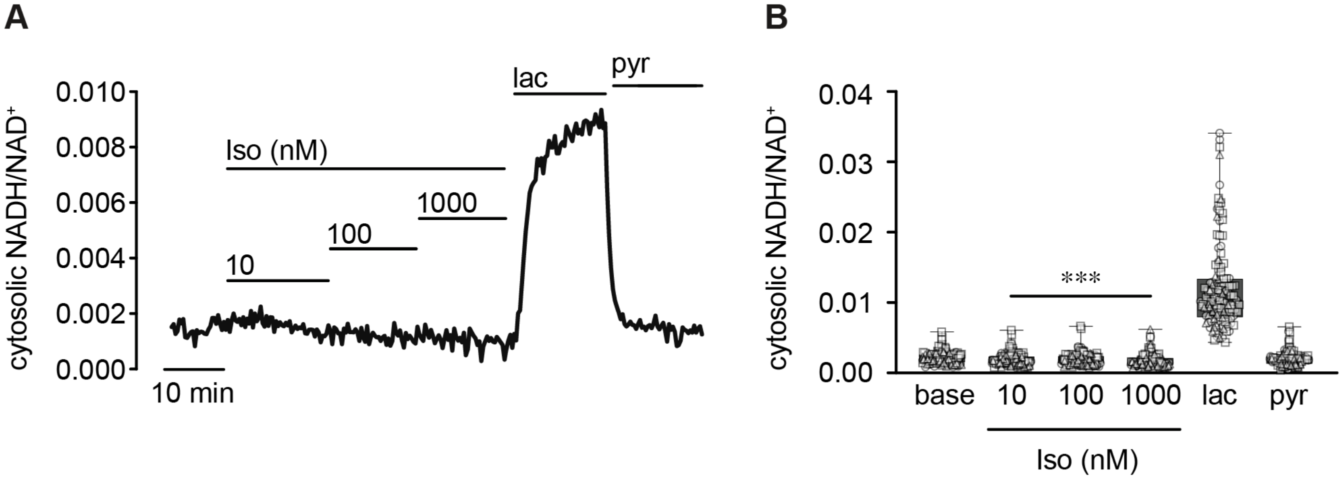

Supplementary Figure 4: NADH:NAD$^+$ is not elevated by direct β-adrenergic stimulation. (**A**) Representative recordings of cytosolic NADH:NAD$^+$ in arterial myocyte before and after application of isoproterenol (iso; 10-1000 nM), prior to application of 10 mM lactate and 10 mM pyruvate at the end of the experiment. (**B**) Box and whiskers plot (line: median, box: 25th to 75th percentile, whiskers: min and max) summarizing NADH:NAD$^+$ at baseline (base) and in the presence of iso at concentrations indicated, or in the presence of 10 mM lactate or pyruvate. N = 123 cells, 3 independent experiments (denoted by symbols). ***p = 0.00001 vs. base (one-way repeated measures ANOVA).

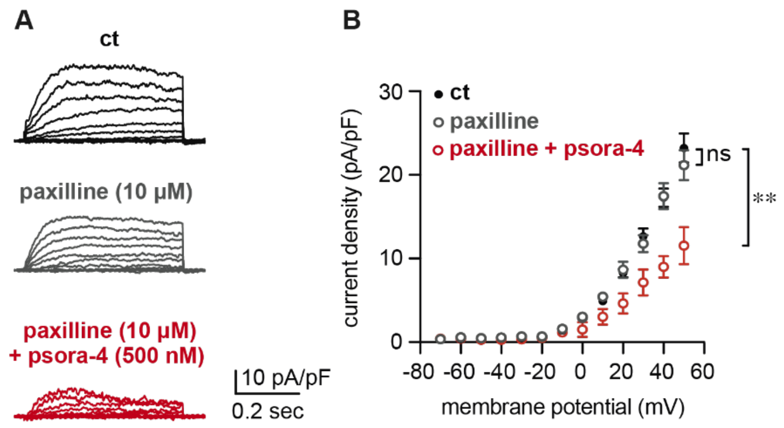

Supplementary Figure 5: Contribution of Kv1 channels to total outward I_K in coronary arterial smooth muscle. (**A,B**) Representative voltage-clamp I_K recordings (normalized to cell capacitance, pA/pF, *A*) and summarized I_K densities (mean values ± SEM, *B*) in coronary arterial myocytes at depolarizing membrane potentials from -70-50 mV in the absence (ct) and presence of the BK_{Ca} inhibitor paxilline (10 µM), or in the presence of paxilline (10 µM) and psora-4 (500 nM). n = 7-9 cells from 4 mice. ns, p = 0.9891, ct vs. paxilline, **p = 0.0006, ct vs. paxilline + psora-4 (mixed effects analysis with Dunnett correction).

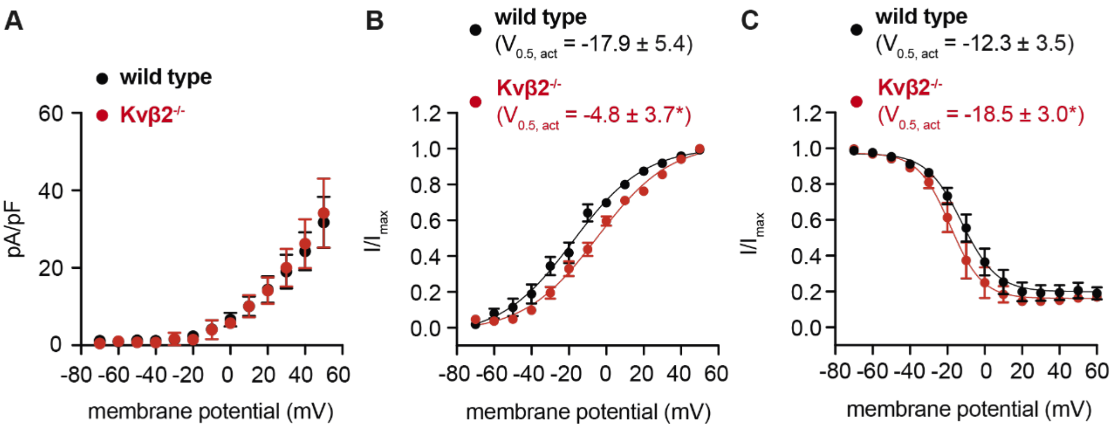

Supplementary Figure 6: Impact of Kvβ2 ablation on whole cell I_K in coronary arterial smooth muscle. (**A**) Summary of total outward I_K density (mean values ± SEM) in freshly isolated coronary arterial myocytes from wild type (129SvEv) and Kvβ2$^{-/-}$ mice. n = 7-8 cells, 4-5 mice for each. (**B, C**) Plots showing summarized I/Imax (mean values ± SEM) across range of membrane potentials from activation and inactivation two-pulse voltage protocols in wild type and Kvβ2$^{-/-}$ mice. Data are fit with Boltzmann function. n = 6-12 cells, 4-5 mice for each. *B:* *p = 0.000013; *C:* *p = 0.008002, Kvβ2$^{-/-}$ vs. wild type (extra sum-of-squares F test).

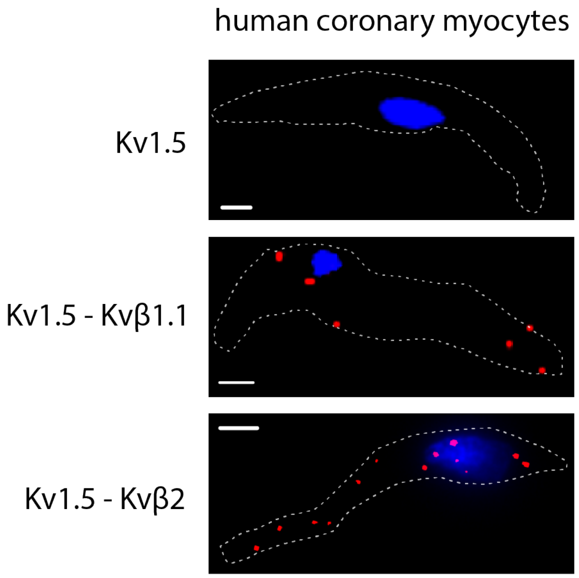

Supplementary Figure 7: Native Kv1 channels in human coronary arterial myocytes associate with Kvβ proteins. Representative fluorescent images showing proximity ligation-positive punctae (red) in isolated human coronary arterial myocytes co-labelled for Kv1.5 and Kvβ1.1, and Kv1.5 and Kvβ2 proteins. As a control, cells were also labelled for Kv1.5 alone. Images are representative of 4, 10, and 14 cells for cells labelled for Kv1.5 alone, Kv1.5 – Kvβ1.1, and Kv1.5 – Kvβ2, respectively.

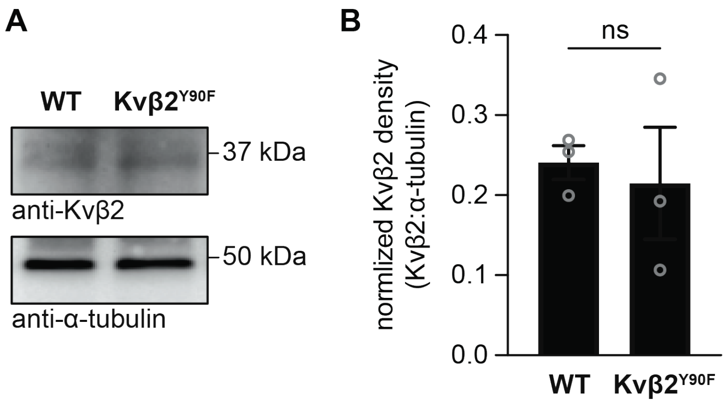

Supplementary Figure 8: The Y90F mutation in Kvβ2 does not alter the abundance of vascular Kvβ2. (**A-B**) Representative blot images showing immunoreactive bands for Kvβ2 and a-tubulin (loading control) (*A*) and summary (mean values ± SEM) of Kvβ2-associated band densities normalized to α-tubulin (*B*) in mesenteric artery lysates from wild type and KvB2^{Y90F} mice. n = 3 each; ns: P>0.05 (two-sided Mann Whitney U test).

Supplementary Table 1: Available clinical information for human coronary arterial tissue.

Sex	Male
Age	52 yrs
Cause of death	Cerebrovascular/stroke
BMI	26.4
**Medical history**	
Diabetes	No
Cancer	No
Coronary artery disease	No
Gastrointestinal disease	No
Chest trauma	No
Cigarette use (>20 pack yr)	Yes
Continued cigarette use (prior 6 mo)	Yes
Heavy alcohol use (≥2 drinks/day)	Yes
I.V. drug use	No
Risk of blood-borne disease transmission	No
COVID-19	Negative

Supplementary Table 2: Pyridine nucleotide concentrations and redox ratios for whole cell I_{Kv} recordings.

	**Oxidized**	**Reduced**
**NAD^+ (μM)**	1000	200
**NADH (μM)**	50	1000
**NADH:NAD^+**	0.05	5
**$NADP^+$ (μM)**	30	50
**NADPH (μM)**	100	100
**NADPH:$NADP^+$**	3.33	2

Supplementary Table 3: Voltage-sensitivity of Kv activation and inactivation.

	**Wild type**		**Kvβ2$^{-/-}$**	
	**Oxidized**	**Reduced**	**Oxidized**	**Reduced**
**$V_{0.5,\ act}$** (*mV*)	-2.5 ± 3.8	-17.5 ± 3.1 (*p = 0.0021)	-11.2 ± 3.0	10.9 ± 6.3 (*p = 0.0017)
**$V_{0.5,\ inact}$** (*mV*)	-35.7 ± 3.1	-49.0 ± 1.4 (*p = 0.0003)	-51.4 ± 2.9	-31.4 ± 4.9 (*p = 0.0133)

$V_{0.5,\ act/inact}$: voltage at 50% of maximum I/Imax, n = 5-7 cells, 4-5 mice for each, *p value, reduced vs. oxidized (extra sum-of-squares F test).

Uncut blot for Supplementary Figure 8

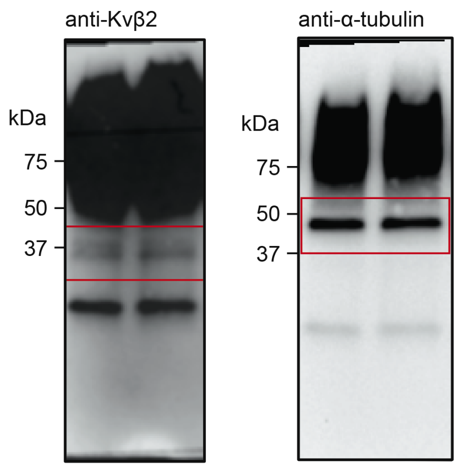

Supplementary References

1. Hung YP, Albeck JG, Tantama M, Yellen G. Imaging cytosolic NADH-NAD(+) redox state with a genetically encoded fluorescent biosensor. *Cell Metab* **14**, 545-554 (2011).

2. Hung YP, Yellen G. Live-cell imaging of cytosolic NADH-NAD+ redox state using a genetically encoded fluorescent biosensor. *Methods Mol Biol* **1071**, 83-95 (2014).
