# Supplementary material for: Pyridine nucleotide redox potential in coronary smooth muscle couples myocardial blood flow to cardiac metabolism
Source: Nat Commun. 2022 Apr 19;13:2051. doi: 10.1038/s41467-022-29745-z (PMC9018695; doi:10.1038/s41467-022-29745-z)
Supplement: Supplementary file 3 — Reporting Summary [file 41467_2022_29745_MOESM3_ESM.pdf]

nature portfolio

Corresponding author(s): Matthew A. Nystoriak

Last updated by author(s): January 27, 2022

Reporting Summary

Nature Portfolio wishes to improve the reproducibility of the work that we publish. This form provides structure for consistency and transparency in reporting. For further information on Nature Portfolio policies, see our Editorial Policies and the Editorial Policy Checklist.

Statistics

For all statistical analyses, confirm that the following items are present in the figure legend, table legend, main text, or Methods section.

n/a	Confirmed	
☐	☒	The exact sample size (*n*) for each experimental group/condition, given as a discrete number and unit of measurement
☐	☒	A statement on whether measurements were taken from distinct samples or whether the same sample was measured repeatedly
☐	☒	The statistical test(s) used AND whether they are one- or two-sided<br>*Only common tests should be described solely by name; describe more complex techniques in the Methods section.*
☐	☒	A description of all covariates tested
☐	☒	A description of any assumptions or corrections, such as tests of normality and adjustment for multiple comparisons
☐	☒	A full description of the statistical parameters including central tendency (e.g. means) or other basic estimates (e.g. regression coefficient) AND variation (e.g. standard deviation) or associated estimates of uncertainty (e.g. confidence intervals)
☐	☒	For null hypothesis testing, the test statistic (e.g. *F*, *t*, *r*) with confidence intervals, effect sizes, degrees of freedom and *P* value noted<br>*Give P values as exact values whenever suitable.*
☒	☐	For Bayesian analysis, information on the choice of priors and Markov chain Monte Carlo settings
☒	☐	For hierarchical and complex designs, identification of the appropriate level for tests and full reporting of outcomes
☒	☐	Estimates of effect sizes (e.g. Cohen's *d*, Pearson's *r*), indicating how they were calculated

Our web collection on statistics for biologists contains articles on many of the points above.

Software and code

Policy information about availability of computer code

Data collection	BZ-X800 Viewer version 01.01.01.03 (Keyence), IonWizard6.6 (IonOptix), Clampex10.2 (Axon), LabChart 8 (ADI)
Data analysis	IonWizard6.6 (IonOptix), BZ-X800 Analyzer 1.1.1.8 (Keyence), ImageJ2.0.0-rc-69/1.53i (NIH), FlexImaging version 5.0.72.0_978_145 (Bruker), Clampfit10.6 (Axon), LabChart 8 (ADI), GraphPad Prism 9, SAS 9.4, IMCE (from Oregon Health & Science University: Lepper et al., Circulation, 2004, Jun 29; 109(25):3132-5, PMID: 15226230; Noble et al., Ultrasound in Medicine and Biology, 2002, Jan, 28(1):115-123, PMID: 11879958.

For manuscripts utilizing custom algorithms or software that are central to the research but not yet described in published literature, software must be made available to editors and reviewers. We strongly encourage code deposition in a community repository (e.g. GitHub). See the Nature Portfolio guidelines for submitting code & software for further information.

Data

Policy information about availability of data

All manuscripts must include a data availability statement. This statement should provide the following information, where applicable:

- Accession codes, unique identifiers, or web links for publicly available datasets
- A description of any restrictions on data availability
- For clinical datasets or third party data, please ensure that the statement adheres to our policy

All data analyzed or generated in this study are included in the main manuscript and/or supplementary figures. Source data are provided in a Source data file.

Field-specific reporting

Please select the one below that is the best fit for your research. If you are not sure, read the appropriate sections before making your selection.

- [x] Life sciences
- [ ] Behavioural & social sciences
- [ ] Ecological, evolutionary & environmental sciences

For a reference copy of the document with all sections, see nature.com/documents/nr-reporting-summary-flat.pdf

Life sciences study design

All studies must disclose on these points even when the disclosure is negative.

Sample size	The number of replicates included in samples was based on previously published datasets using similar methods required to reach statistical differences between groups (Ohanyan et al., Circ Res. 2021. Mar. 19; 128(6):738-751, PMID: 33499656; Kilfoil et al., J Mol Cell Cardiol. 2019, Dec. 137:93-106; Nystoriak et al., Sci Signaling, 2017. Jan 24: 10(463):eaaf9647; Nystoriak et al., Circ Res., 2014, Feb 14; 114(4):607-15)
Data exclusions	No data were excluded.
Replication	Reproducibility was verified by performing experiments to generate datasets from cells/tissues from at least two mice. Attempts at replication were successful.
Randomization	Mice were randomly assigned to either low cardiac work and high cardiac work groups for experiments shown in Figure 1. For all other experiments, randomization was not relevant since experimental treatments were performed ex vivo, in vitro.
Blinding	Blinding for some experiments was not possible due to the experimental design (e.g., acute treatments). Project personnel performing all in vivo measurements of MAP, HR, and MBF were blinded to mouse genotype.

Reporting for specific materials, systems and methods

We require information from authors about some types of materials, experimental systems and methods used in many studies. Here, indicate whether each material, system or method listed is relevant to your study. If you are not sure if a list item applies to your research, read the appropriate section before selecting a response.

Materials & experimental systems

n/a	Involved in the study	
[ ]	[x]	Antibodies
[ ]	[x]	Eukaryotic cell lines
[x]	[ ]	Palaeontology and archaeology
[ ]	[x]	Animals and other organisms
[ ]	[x]	Human research participants
[x]	[ ]	Clinical data
[x]	[ ]	Dual use research of concern

Methods

n/a	Involved in the study	
[x]	[ ]	ChIP-seq
[x]	[ ]	Flow cytometry
[x]	[ ]	MRI-based neuroimaging

Antibodies

Antibodies used	Mouse monoclonal anti-Kvβ2 (Neuromab 75-021; clone K17/70; 1:400 in WB)<br>Mouse monoclonal anti-α-tubulin (clone B-5-1-2; Sigma Aldrich, T5168, 1:4000 in WB)<br>Mouse anti-Kv1.5 (Neuromab, 75-011, 1:50 in proximity ligation assays)<br>Rabbit polyclonal anti-Kvβ1 (Abcam, Ab174508, 1:100 in proximity ligation assays)<br>Rabbit polyclonal anti-Kvβ2 (Aviva Systems Biology, ARP37678-t100, 1:100 in proximity ligation assays)<br>Horse anti-mouse IgG, HRP-linked (Cell Signaling Technology, 7076, 1:3000 in Western blot).
Validation	Anti-Kvβ2 (Neuromab and Aviva), anti-Kvβ1 (Abcam), and anti-Kv1.5 (Neuromab) antibodies were validated in-house via confirmation of signal only in Cos-7 cells expressing target protein of interest, or loss of signal in cells from knockout mice. See Kilfoil et al., J Mol Cell Cardiol., 2019, Dec; 137:93-106, PMID: 31639389; Nystoriak et al., Chem Biol Interact., 2017, Oct 1; 276:210-217, PMID: 28342889.<br><br>Anti-α-tubulin - (https://www.sigmaaldrich.com/US/en/product/sigma/t5168?gclid=Cj0KCQiAtJeNBhCVARIsANJUJ2Huj1GAc5LWnZ910O7hSE0_79Y7N1FSzFRUk7dmD0kekmgX9-awZ0waAvOXEALw_wcB)

Eukaryotic cell lines

Policy information about cell lines

Cell line source(s)	Cos-7 cells were from Sigma Aldrich (87021302; Lot: 15I032)
Authentication	Lot was tested for cell count, viability, and confluency upon resuscitation, detection of myoplasma (PCR and Vero indicator cell line and Hoechst 33258 fluorescence detection), speciation (DNA bar-coding sequencing of the mitochondrial cytochrome c oxidase (COX) subunit 1 gene), and sterility.
Mycoplasma contamination	Cells were negative for mycoplasma contamination.
Commonly misidentified lines (See ICLAC register)	None.

Animals and other organisms

Policy information about studies involving animals; ARRIVE guidelines recommended for reporting animal research

Laboratory animals	Male age-matched (3-6 mo) mice in which Kcnab1 or Kcnab2 genes were ablated or in which Tyrosine 90 of Kvβ2 was mutatedd to Phenylalanine were used for this study. Background strain-matched wild type mice (C57Bl6N for Kvβ1.1-/-, 129SvEv for Kvβ2-/-) were used as controls. All mice were bred and maintained in-house and fed normal chow ad libitum in a temperature-controlled room on a continuous 12:12 h light:dark cycle.
Wild animals	No wild animals were used in this study.
Field-collected samples	No field-collected samples were used in this study.
Ethics oversight	All animal procedures were performed as approved by the Institutional Animal Care and Use Committees at the University of Louisville and Northeast Ohio Medical University.

Note that full information on the approval of the study protocol must also be provided in the manuscript.

Human research participants

Policy information about studies involving human research participants

Population characteristics	Human tissue from a single donor was used in our study. Covariate-relevant characteristics are provided in Table S1 (see Supplemental Data.
Recruitment	Recorded phone authorization for anatomical gift.
Ethics oversight	Authorization under Uniform Anatomical Gift Act. No IRB approval is required since the work does not meet the common rule definition of human subjects research.

Note that full information on the approval of the study protocol must also be provided in the manuscript.
